# SOD2 ameliorates pulmonary hypertension in a murine model of sleep apnea via suppressing expression of NLRP3 in CD11b^+^ cells

**DOI:** 10.1186/s12931-019-1270-0

**Published:** 2020-01-08

**Authors:** Cuiping Fu, Shengyu Hao, Zilong Liu, Liang Xie, Xu Wu, Xiaodan Wu, Shanqun Li

**Affiliations:** Department of Pulmonary Medicine, Zhongshan Hospital, Fudan University, 180 Fenglin Road, Shanghai, 200032 China

**Keywords:** Chronic intermittent hypoxia, Pulmonary hypertension, SOD2, NLRP3, CD11b

## Abstract

**Background:**

High prevalence of obstructive sleep apnea (OSA) in the pulmonary hypertension (PH) population suggests that chronic intermittent hypoxia (CIH) is an important pathogenic factor of PH. However, the exact mechanism of CIH induced PH is not clear. One of the molecules that plays a key role in regulating pulmonary artery function under hypoxic conditions is superoxide dismutase 2 (SOD_2_).

**Methods:**

Our study utilized heterozygous SOD_2_^−/+^ mice firstly in CIH model to explore the exact role of SOD_2_ in CIH causing PH. Expression of SOD2 was analyzed in CIH model. Echocardiography and pulmonary hypertension were measured in wild type (WT) and SOD2^−/+^ mice under normal air or CIH condition. Hematoxylin–Eosin (H&E) staining and masson staining were carried out to evaluate pulmonary vascular muscularization and remodeling. Micro-PET scanning of in vivo ^99m^Tc-labelled- MAG3-anti-CD11b was applied to assess CD11b in quantification and localization. Level of nod-like receptor pyrin domain containing 3 (NLRP3) was analyzed by real time PCR and immunohistochemistry (IHC).

**Results:**

Results showed that SOD_2_ was down-regulated in OSA/CIH model. Deficiency of SOD2 aggravated CIH induced pulmonary hypertension and pulmonary vascular hypertrophy. CD11b^+^ cells, especially monocytic myeloid cell line-Ly6C^+^Ly6G^−^ cells, were increased in the lung, bone marrow and the blood under CIH condition, and down-regulated SOD2 activated NLRP3 in CD11b^+^ cells. SOD_2_-deficient-CD11b^+^ myeloid cells promoted the apoptosis resistance and over-proliferation of human pulmonary artery smooth muscle cells (PASMCs) via up-regulating NLRP3.

**Conclusion:**

CIH induced down-regulating of SOD2 increased pulmonary hypertension and vascular muscularization. It could be one of the mechanism of CIH leading to PH.

## Introduction

Obstructive sleep apnea (OSA) is a public health problem in 5–10% people, which causes episodic hypoxemia and elevates blood pressure and markers of oxidative stress, inflammation, and hypercoagulation [1–3]. Several studies have reported OSA as a risk factor for systemic hypertension and various cardiovascular diseases [4, 5]. Pulmonary hypertension (PH) was the most common cause of death in cardiovascular complications. Most authors claim that nocturnal apnea cannot induce permanent PH and that PH in patients with OSA is related to an associated obstructive ventilatory defect [6]. However, a few recent studies have produced different results which suggest a direct link between OSA and daytime PH. PH in patients with OSA predicts functional limitations and a poor prognosis [7].

Chronic intermittent hypoxia (CIH) is a key character of OSA, which can lead to PH and other cardiovascular diseases. Exaggerated negative intrathoracic pressure during obstructive apneas is the main contributing factor of PH. Sleep disordered breath (SDB) was present in 71% of the PH patients: 56% had OSA and 44% (central sleep apnea) CSA [8]. Older age and subjective sleepiness as assessed by the Epworth Sleepiness Scale score > 10 were predictive of SDB. A high prevalence of OSA occurred in both male (50%) and female (60%) subjects. This high prevalence of OSA in the PH population suggests that CIH is an important pathogenic factor of PH. However, the exact mechanism of CIH induced PH is not clear. Further studies are necessary to determine the relationship between OSA and PH.

Several studies indicated that CIH led to inhibition of superoxide dismutase-2 (SOD_2_) transcription, resulting in oxidative stress. CIH raised reactive oxygen species levels along with a decrease in SOD_2_, an anti-oxidant enzyme. Manganese superoxide dismutase (MnSOD) in the mitochondria plays an important role in cellular defense against oxidative damage [9]. Homozygous MnSOD knockout (Sod_2_^−/−^) mice are neonatal lethal, indicating the essential role of MnSOD in early development, however, it limited the exploration of SOD_2_ to some degree [10, 11]. Over-expression of transcriptionally active HIF-2α prevented CIH-evoked oxidative stress and restored SOD2 activity, and thus attenuated CIH induced lung injury. SOD_2_ was also found to be decreased in PH model [12]. One of the molecules that plays a key role in regulating pulmonary artery function under hypoxic conditions is SOD_2_. There is significant association between expression of SOD_2_ and the PH susceptibility [13]. Our previous study proved that wild type (WT) mice progressed into PH after 5-week CIH [14]. We speculated that SOD_2_ played an important role in CIH induced PH. Our study utilized heterozygous SOD_2_^−/+^ mice firstly in CIH model to explore the exact role of SOD_2_ in CIH causing PH.

Decreased expression of SOD_2_ was related to the function of myeloid cells and thus led to various kinds of inflammation. Downregulation of SOD_2_ gene expression in neutrophils caused neutrophil dysfunction and cytokine dysregulation, reduced expression of interleukin (IL) 1A, IL-1R1, IL-1R2 and IL8RA gene expression, which leading to chronic kidney disease [15]. Reduced expression of SOD_2_ in CD11b^+^Gr1^+^ cells participated in the initiation and progression of leukemia [16]. Intracellular SOD_2_ has a protective role by suppressing Nod-like receptor pyrin domain containing 3 (NLRP3) inflammasome-caspase-1-IL-1β axis under inflammatory conditions [17]. NLRP3 inflammasome participated in the differentiation in and/or recruitment to gastrointestinal, lung, and lymphoid tissues of CD11b^+^ dendritic cells. Studies have shown that NLRP3 inflammasome contributes to PH pathogenesis. Intriguingly, the inhibition of the NLRP3-inflammasome pathway ameliorates PH, which was induced by monocrotaline, a substance largely used in experimental set-up to produce PH [18].

The pathophysiological mechanism of PH was reported to be related to myeloid development and immune regulation [19]. Our study focused on the myeloid immune response and tried to find out the mechanism of CIH induced PH. We assumed that CIH down-regulated expression of SOD_2_ regulated the differentiation of CD11b^+^ myeloid cells, which caused a series of oxidative stress response, including NLRP3 inflammasome, and thus resulted in PH. In this study, we are trying to explore the role of SOD_2_ in CIH induced PH model by using SOD_2_-defected mice, pulmonary molecular mechanisms involved, specially NLRP3, from the aspect of myeloid immune response about how SOD_2_ act on vascular remodeling under CIH condition as a possible explanation for OSA-induced pulmonary hypertension.

## Methods

### Human samples

This study was ethic approved by the Institutional Review Board of Zhongshan Hospital and written informed consent was obtained from all patients. Blood were obtained from patients diagnosed with OSA in sleep center of Shanghai Zhongshan Hospital affiliated to Fudan University and healthy donators.

### Experimental animals

Mice were maintained and bred in Fudan University animal facility according to the National Institutes of Health Guidelines for the Humane Treatment of Laboratory Animals. All animal procedures have been approved by Fudan University institutional animal care and use committee in accordance with the Helsinki Declaration of 1975. Because Sod_2_^−/−^ mice are neonatal lethal, heterozygous SOD2^−/+^ mice were adopted in this study. C57bl/6 and SOD_2_^−/+^ mice were obtained as described from Nanjing model animal center [20, 21]. Mice were maintained on a 12:12- h night-day cycle, with standard mice chow and water available ad libitum. All experiments were conducted 6-week-old male mice in pathogen-free facilities.

### CIH model

The animals were weighed and randomly divided into two groups. One group was exposed to CIH. The other group was exposed to air and used as a control. An established rodent model of CIH was utilized [22]. Briefly, CIH mice were placed into a specially designed chamber, which contained a gas control delivery system to regulate the flow of oxygen and nitrogen into the chamber. During each 1-min period of intermittent hypoxia, the oxygen concentration in the chamber was adjusted between 5 and 21%. Nitrogen was introduced at a rate sufficient to achieve a fraction of inspired oxygen (FiO2) of 5% within 30 s and to maintain this level of FiO2 for 10 s; then, oxygen was introduced at a rate to achieve a FiO2 of 21% within 20 s. C57bl/6 mice were placed into this chamber for 9 h daily, 7 days per week, for 6 consecutive weeks. At all other times, the mice were kept in chambers with an oxygen concentration of 21%. The oxygen concentration in these chambers was continuously observed by an oxygen analyzer, and the levels were under feedback control by a computerized system connected to a gas valve outlet. Deviation from the determined settings was corrected by the addition of pure nitrogen or oxygen through solenoid valves. The control group was handled in the same manner as the CIH group, except oxygen concentration in the control chambers was maintained at a constant 21% throughout the experiment. The experimental protocol was approved by the local Animal Care and Use Committee of Fudan University.

### Echocardiography

Echocardiographic measurement is a valuable parameter to reflex the degree of PH. Briefly, mice were slightly anesthetized using isoflurane and analyzed within 2 h. Hair of the mice were moved for better scanning. Inhaled isoflurane was administered at 3% induction and 1–1.5% maintenance. After anesthetization, each mice was placed in the supine position on a temperature-controlled pad. All these echocardiography was handled by the skilled animal technician in animal heart ultrasonic laboratory. The probe of a Vivid S5 echocardiography system (Vivid S5, GE Healthcare, Chicago, IL) was gently be tilted laterally to obtain a view of the PH crossing over the aorta. After that, the ultrasound was switched to the color Doppler mode, parallel to the direction of blood flow in the vessel to obtain a flow waveform. Right ventricle cross-sectional area (RVCSA) and tricuspid annular plane systolic excursion (TAPSE), parameters correlating well with right ventricular function, were collected for further analysis.

### PH pressure measurement

Right ventricular systolic pressure (RVSP), conventionally used as an indicator of mean pulmonary arterial pressure, was measured by closed-chest puncture of the right ventricle (RV). After measurement through echocardiography, a longitudinal skin incision was made on the right side of the neck, and blunt layer-by layer separation of the tissues was performed until the right external jugular vein was exposed. A polyethylene catheter was gradually inserted into the pulmonary artery through an incision in the right external jugular vein, and the RV systolic pressure was recorded using a pressure transducer, which was interfaced to a BL-420S Bio Lab System (Chengdu TME Technology Co., Ltd., Chengdu, China). At the end of the experiment, the mice were anesthetized with an intraperitoneal injection of 3% sodium pentobarbital, and various organs were harvested. RV hypertrophy was evaluated as the ratio of the weight of the RV wall to that of the left ventricle plus septum.

### Expression analyses

For RNA extraction and quantitative real time-polymerase chain reaction (RT-PCR) analysis, total RNA was extracted from tissues using Trizol (Invitrogen, California, USA). High fidelity cDNA was generated from each RNA samples with Superscript III cDNA amplification System (Invitrogen). Quantitative RT-PCR reaction samples were prepared as a mixture with Quantitect SYBR Green PCR kit (Qiagen, Dusseldorf, Germany) following the manufacturer’s instructions. Reactions were performed using an Applied Biosystems Prism 9700 PCR machine. The PCR conditions were as follows: 95 °C for 30 s followed by 45 cycles of 95 °C for 15 s, 55 °C for 30 s and 72 °C for 30 s. For protein analysis, Western blot was carried out as previous described [23]. SOD_2_ antibody was purchased from abcam Co. USA.

### Hematoxylin–eosin (H&E) staining and immunohistochemical (IHC) analyses

Formalin-fixed paraffin-embedded tissue sections were dewaxed, hydrated, heated for 10 min in a conventional pressure cooler, treated with 3% H_2_O_2_ for 30 min, and then incubated with normal fetal bovine serum for 30 min. Sections were then incubated with antibodies overnight (anti-NLRP3 diluted 1:200) after washing and then were incubated with biotin-labeled secondary antibody for 60 min at 37 °C after washing. The multiplicative quick score method (QS) were applied to assess the intensity and the extent of cell staining of NLRP3. In brief, the proportion of positive cells was estimated and given a percentage score on a scale from 1 to 6 (1 = 1–4%; 2 = 5–19%; 3 = 20–39%; 4 = 40–59%; 5 = 60–79%; and 6 = 80–100%). The average intensity of the positively staining cells was given an intensity score from 0 to 3 (0 = no staining; 1 = weak, 2 = intermediate, and 3 = strong staining). The QS was then calculated by multiplying the percentage score by the intensity score to yield a minimum value of 0 and a maximum value of 18. HE staining was conducted as previously described [24]. Masson staining was conducted as previously described [14].

### Antibodies and fluorescence activating cell sorter (FACS) analysis

PE-conjugated lineage specific antibodies (Ly6G), APC-conjugated CD11b antibody, PercPcy5.5-conjugated Ly6C were used in FACS (BD Pharmingen, USA). For FACS analysis, isolated cells were washed with phosphate-buffered saline and then incubated with anti-mouse antibody described above for 40 min at 4 °C to achieve specific binding. Single-cell suspensions were evaluated by multi-color flow cytometry using a LSRII flow cytometer (BD Biosciences). Data was analyzed using FlowJo 10 software (BD bioscience Inc. USA).

### Micro-PET scanning of in vivo ^99m^Tc-labelled- MAG3-anti-CD11b

Antibody CD11b modification was followed by previous described [25]. MAG3-anti-CD11b was labeled with ^99m^Tc following the developed method [25]. Briefly, 50 μl lgMAG3-anti-CD11b conjugate was added to a solution of mixed 45 μl of 0.25 M ammonium acetate and 15 μl tartrate buffer, getting rid of physical movement, and then ^99m^Tc-pertechnetate generator eluate was added. Using acetone as the developing solvent, the labeling rate was measured by radio- TLC (Bioscan, Washington, DC, USA): 99mTc-MAG3-anti-CD11b antibody, Rf = 0; Na99mTcO4, Rf = 0.73. PD-10 columns were used for products purification with 0.25 M ammonium acetate as eluent, and the first radioactive product peak was recorded. Mice were prepared for SPECT/CT scanning. The parameter of FOV (Transaxial) is 80 mm, spatial resolution is less than 1.3 mm, FOV (Axial) is 60 mm, peak value is 226Kcps and sensitivity is 4.3% (380~640Kev), content rate of scattered radiation is 6 .3%.

### CD11b^+^ cells and co-culture system

CD11b^+^ cells were separated from C57BL/6 and SOD_2_^−/+^ mice using magnetic beads. Bone marrow cells and lung cells from C57BL/6 and SOD_2_^−/+^ mice was collected and made into single-cell suspensions. Anti-CD11b magnetic microbeads were used to obtain CD11b^+^ cells.

### Brdu detection

Brdu staining in this study was applied to measure the level of cell proliferation. Cells were covered with FBS for 3 days and then treated with Brdu (20 mM, CAT:558599) for 1 h in 37 C, and then washed. Cells in the plates were then treated with paraffin and incubated in HCl (1 M) for 10 min on ice, followed by HCL (2 M) for 10 min at room temperature and 20 min at 30 C. Neutralize by borate buffer solution 10 min at room temperature. After washed with phosphate buffer solution for three to five times, cells was observed by immunofluorescence microscope for brdu positive staining.

### Antibodies and immunofluorescence staining

The following antibodies were used in current studies: CD11b antibody (abcam, Cambridge, USA); NLRP3 antibody (abcam, Cambridge, USA); Tissue samples from SOD_2_^−/+^/WT mice were fixed with 4% paraformaldehyde for 12 h followed by 30% sucrose overnight. Texas Red -conjugated rabbit-specific secondary antibody and FITC-conjugated mouse-specific secondary antibody (Biolegend, USA) were used for immunofluorescence analysis of NLRP3 and CD11b respectively. NLRP3 and CD11b fluorescence expression were observed and captured by Leica TCS SP8 (Germany).

### Statistical analyses

Data from at least three independent experiments or five mice per group are represented as mean ± SD. Normality distribution was measured before using unpaired Student’s t tests. Comparisons between multiple groups were performed using ANOVA with the Bonferroni test. A *P* value < 0.05 was considered statistically significant. * meant *p* < 0.05; ** meant *p* < 0.01; ***meant *p* < 0.001.

## Results

### SOD2 was down-regulated in OSA/CIH model

**Fig. 1:**
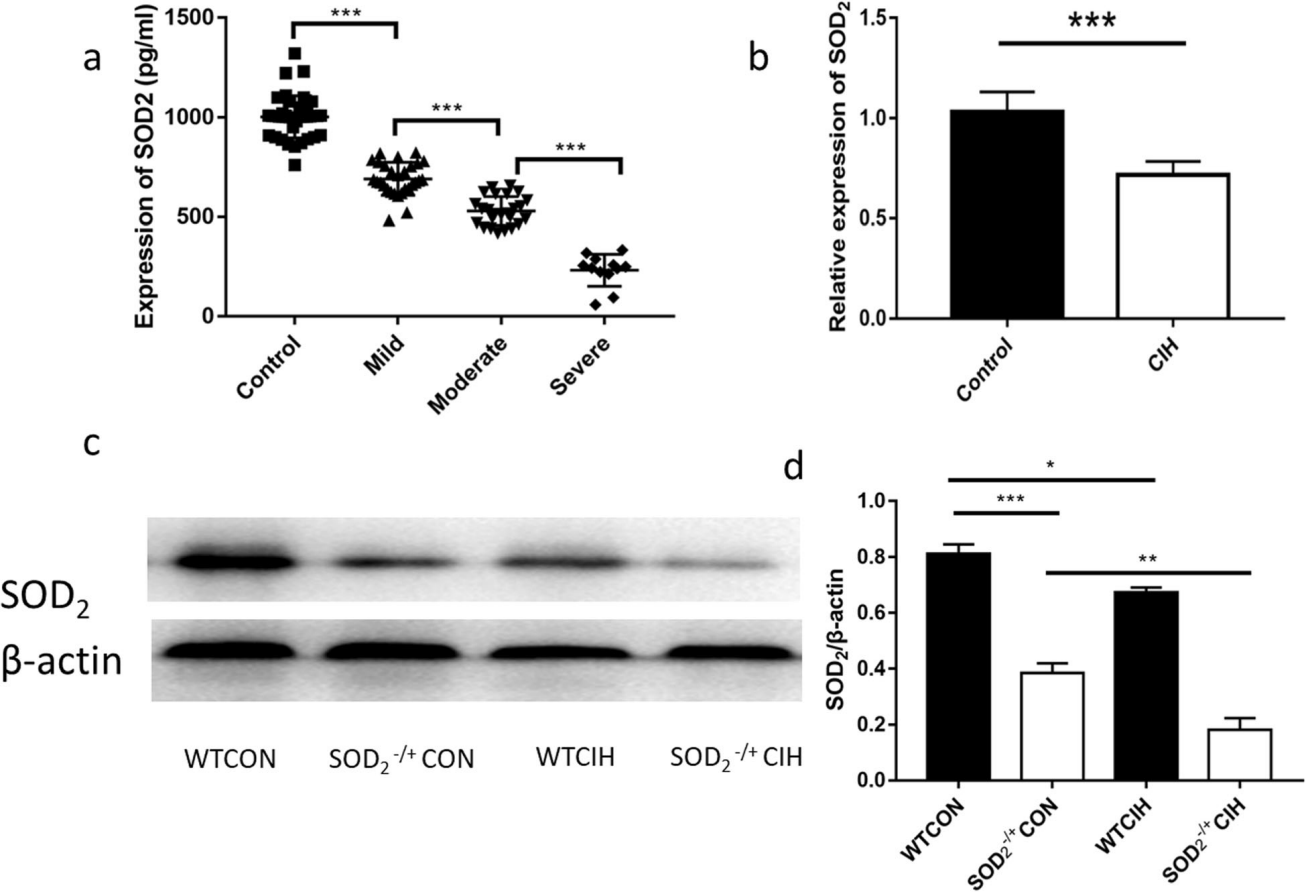
Expression of SOD_2_ in the plasma. **a**. Concentration of SOD_2_ in the plasma of both healthy donators (control) and OSA patients (mild, moderate, severe OSA). **b**. Relative mRNA expression of SOD_2_ in the control and chronic intermittent hypoxia (CIH) mice. **c**. Representative graph of western blot expression of SOD2 compared to β-actin. **d**. Ratio of SOD_2_/β-actin in the group of wild type (WT) mice and SOD2 knockdown mice under control or CIH condition. CON: Wild type mice in the normal air condition. CIH: Chronic intermittent hypoxia. WT: wild type mice. SOD_2_^−/+^: knock-down of SOD_2_ gene heterozygous mice. *N* = 5 in experiment of b and c. Comparisons between two groups were performed using unpaired T-test after normality distribution test. Comparisons between multiple groups were performed using ANOVA with the Bonferroni test.*p < 0.05, **p < 0.01, ***p < 0.001

We analyzed the expression of SOD2 of 117 participants in the blood to compare the deficiency of SOD2 in OSA groups and the control group. Blood samples were obtained from total 117 donators. Among them, 39 samples are from healthy donators, and 29 are diagnosed with mild OSA, 31 with moderate OSA, 18 with severe OSA. Male participants are more than the female in the group of OSA compared to the control group, while no difference in the age (Table 1). SOD2 was found to be decreased in the plasma in OSA patients, and the difference amplified expression of SOD2 was decreased in CIH mice (Fig. 1b), and the expression of SOD2 molecular in the blood was also decreased (Fig. 1c, d). We used heterozygous SOD2-/+ mice in this study, in which SOD2 expression was knocked down.

**Table 1:** Clinical parameter of OSA patients

Severity ^a^	All	(1)Normal	(2)Mild	(3)Moderate	(4)Severe	*P* value
Patient No.(%)	117(100%)	39	29	31	18	
Female	30	8	10	7	5	N VS MID:0.197, 0.03; N vs. MOD:0.834; N vs. S:0.543, MID vs. MOD:0.307; MID vs. S: 0.632 MOD vs. S:0.683;
Male	87	31	19	24	13	
Age	49.58 ± 12.24	40.34 ± 12.47	41.34 ± 22.2	39.26 ± 13.21	47.25 ± 17.36	N VS MID:0.092; N vs. MOD:0.01; N vs. S:0.124, MID vs. MOD:0.11; MID vs. S: 0.674 MOD vs. S:0.526;
BMI	26.58 ± 3.75	29.24 ± 4.21	31.35 ± 5.24	32.46 ± 4.44	36.29 ± 4.29	N VS MID:0.543; N vs. MOD:0.231; N vs. S:0.044, MID vs. MOD:0.61; MID vs. S: 0.034 MOD vs. S:0.031;
AHI(PSG)	12.3 ± 18.24	1.75 ± 1.5	8.24 ± 2.45	20.66 ± 3.71	50.3 ± 18.06	N VS MID:0.052; N vs. MOD:0.02; N vs. S:0.034, MID vs. MOD:0.012; MID vs. S: 0.0044 MOD vs. S:0.016;
ESS	8.28 ± 5.52	4.16 ± 2.97	11.73 ± 0.96	15.75 ± 1.74	26.67 ± 5.5	N VS MID:0.047; N vs. MOD:0.03; N vs. S:0.004, MID vs. MOD:0.001; MID vs. S: 0.213 MOD vs. S:0.025;

a Normal (N): AHI < 5; Mild (MID): 5≦AHI < 15; Moderate (MOD): 15≦AHI < 30; Severe (S):AHI≧30. Clinical parameter including age, gender, body mass index (BMI), apnea hypopnea index (AHI) and epworth sleepiness scale (ESS) score were displayed in the table. The results are shown as mean ± SD. Rank sum test was used for gender difference analysis. One-way anova or kruskalwallis, as appropriate according to the distribution of the data

### SOD2 deficiency modulates CIH induced PH

**Fig. 2:**
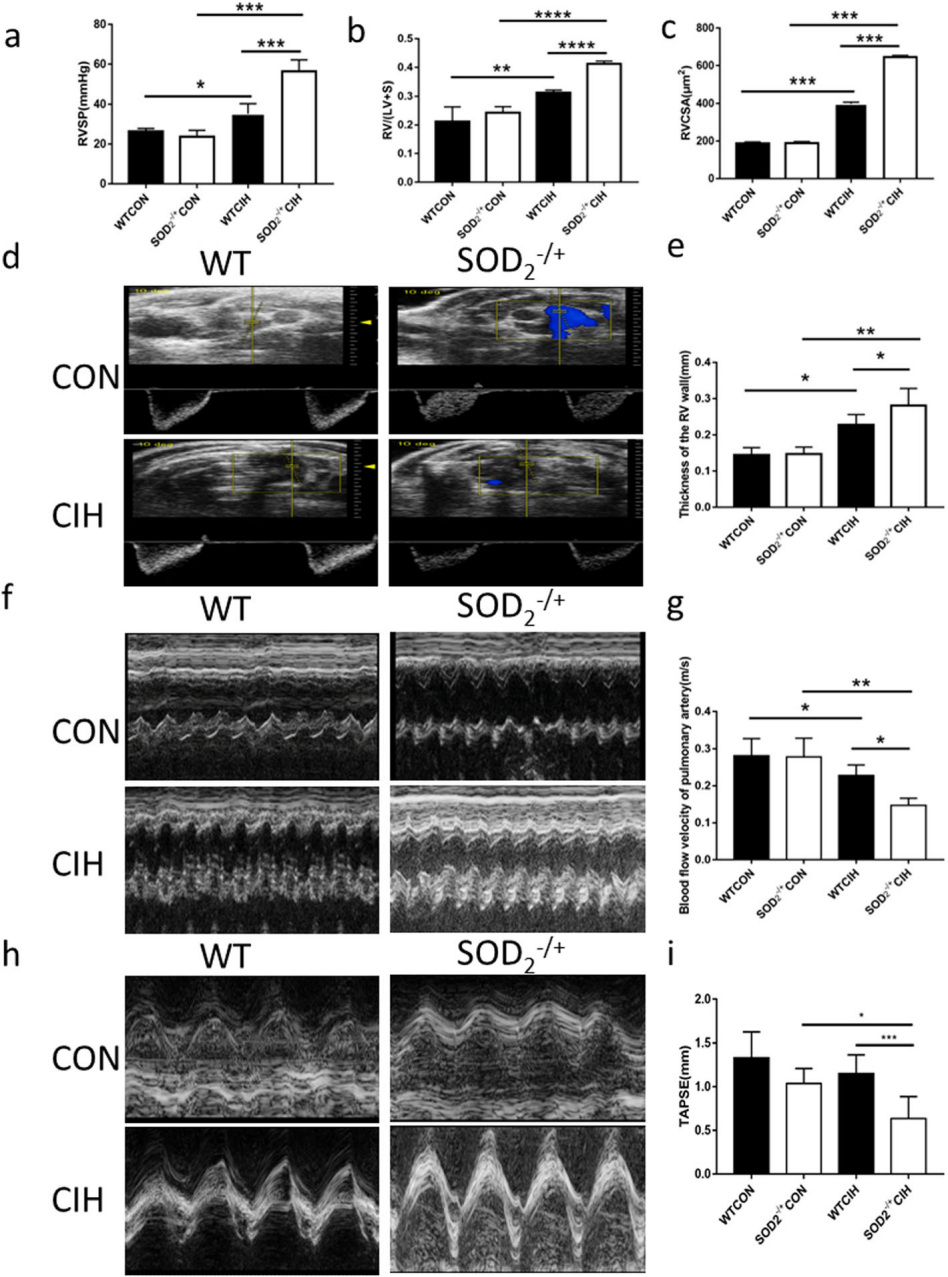
PH pressure detection and Echocardiography in WT and SOD2^−/+^ mice. **a**. Right ventricular systolic pressure (RVSP) measured by closed-chest puncture of the right ventricle (RV). **b**. Ratio of right ventricle to left ventricle plus septum in the four groups. **c**. Statistical analysis of right ventricle cross-sectional area (RVCSA). **d**. Representative enchocardiography of RV. **e**. Statistical analysis of thickness of RV. **f**. The representative view of PA in color Doppler mode, used to assess flow through the PA, was presented. **g**. The blood flow velocity of PA was measured by echocardiography. **h**. The representative enchocardiography of tricuspid annular plane systolic excursion (TAPSE) in the four groups. **i**. Data of TAPSE. CON: Wild type mice in the normal air condition. CIH: Chronic intermittent hypoxia. WT: wild type mice. SOD_2_^−/+^: knock-down of SOD_2_ gene heterozygous mice. *N* = 5. Comparisons between multiple groups were performed using ANOVA with the Bonferroni test. *p < 0.05, **p < 0.01, ***p < 0.001

Six-week of CIH successfully induced PH just as our study declared before. In adult mice, RVSP was higher in SOD2-/+ by exposure to CIH for 6 weeks than WT mice (Fig. 2a, Additional file 1: Figure S1). Right ventricle hypertrophy index (ration of right ventricle (RV) to the left ventricle (LV) and septum (S)) in SOD2-/+ mice were significantly increased compared to those in WT mice (Fig. 2b). Echocardiographic analysis showed elevation in RVCSA in SOD2 knock-down mice, suggesting that diastolic dysfunction resulted from PH (Fig. 2c). Transthoracic echocardiography showed that exposure to CIH further increased the RV wall thickness (Fig. 2d, e), while transthoracic echocardiography confirmed this and also revealed further reduction of the peak velocity of blood flow in the pulmonary artery (Fig. 2f, g). The changes were exacerbated in the SOD2-/+ mice compared with the WT mice following exposure to CIH (Fig. 2d-g). TAPSE correlates well with other standardized parameters of right ventricular function. Echocardiographic analysis in this study showed that TAPSE was attenuated in SOD2-/+ mice compared to WT mice (Fig. 2h-i). Collectively, these findings showed that CIH induced PH with a relative RV hypertrophy in adult mice, and deficiency of SOD2 aggravated CIH by affecting RVSP, ratio of RV/(LV+S), RVCSA, thickness of the RV, blood velocity of pulmonary artery and TAPSE, which might lead to PH.

### SOD2 alleviates pulmonary artery hypertrophy

**Fig. 3:**
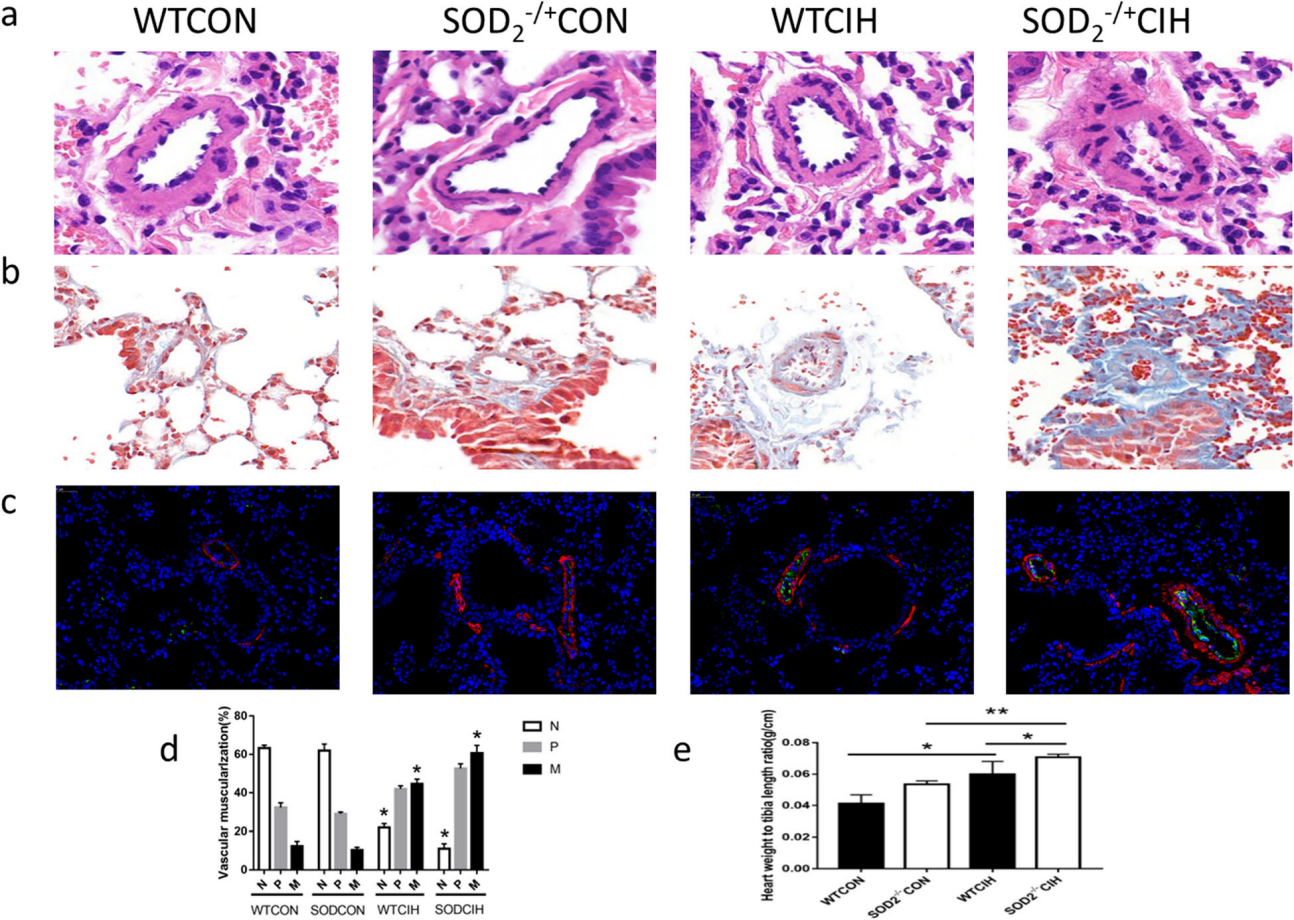
Deficiency of SOD_2_ affects the pulmonary vascular remodeling. **a**. HE staining of pulmonary arterioles in WT and SOD_2_^−/+^ mice. **b**. Masson staining of collagen in WT and SOD_2_^−/+^ mice under CIH condition. **c**. Co-Immunofluorescence staining of the molecular of α-SMA and VWF. α-SMA was stained as red and VWF was green. Cellular nucleus was stained by DAPI (blue). **d**. The same lung lobe from WT and SOD_2_^−/+^ mice were analyzed of pulmonary vessel muscularization. Percentage of area of α-SMA^+^/(α-SMA^+^+VWF^+^) indicated the muscularization degree. Zero-20% represented for no muscularization (N), 20–40% for partly muscularization (P), and 40–100% for fully muscularization (M). **e**. Heart weight to tibia length ration showed the degree of myocardial hypertrophy in WT and SOD_2_^−/+^ mice. CON: Wild type mice in the normal air condition. CIH: Chronic intermittent hypoxia. WT: wild type mice. SOD_2_^−/+^: knock-down of SOD_2_ gene heterozygous mice. *N* = 5. Comparisons between multiple groups were performed using ANOVA with the Bonferroni test. *p < 0.05, **p < 0.01, ***p < 0.001

It is well known that remodeling of pulmonary blood vessels remains to be the main character of PH. Morphometric analyses of lung tissues showd by H&E staining of the pulmonary arterioles indicated that CIH induced thickness of vessel wall compared with the mice in the control group, whereas knock-down of SOD2 aggravated the CIH‐induced morphometric changes in the pulmonary arterioles (Fig. 3a). The hyperplasic state of collagen was observed by Masson collagen stain. Collagen volume fraction was greater in the CIH group, furthermore, this change was amplified in SOD2-/+ group (Fig. 3b). α‐SMA is a positive marker of smooth muscle cells and von Willebrand factor (VWF) is a key molecular in blood vessel formation. Double-immunofluorescence staining of α‐SMA and VWF was shown in Fig. 3c to indicate the classification of vessels muscularized. Percentage of area of α‐SMA+/(α‐SMA++VWF+) indicated the muscularization degree. Zero-20% represented for no muscularization (N), 20%-40% for partly muscularization (P), and 40%-100% for fully muscularization (M). Pulmonary vessel mascularization was showed in Fig. 3d statistically. CIH induced pulmonary vessel muscularization, and deficiency of SOD2 magnified this muscularization in pulmonary arterioles. Heart weight to tibia length ration showed the relative myocardial hypertrophy after deficiency of SOD2 (Fig. 3e). Medial vascular wall thickness was higher in the group of SOD2-/+ mice compared to WT mice under CIH condition showed by elastica van gieson staining (Additional file 1: Fig S2). Taken together, SOD2 could be an important factor of attenuating CIH‐induced pulmonary vessel muscularization, collagen hyperplasia, and smooth muscle progression in the lung.

### Deficiency of SOD2 increased CD11b+ cells to the lung in CIH induced PH

**Fig. 4:**
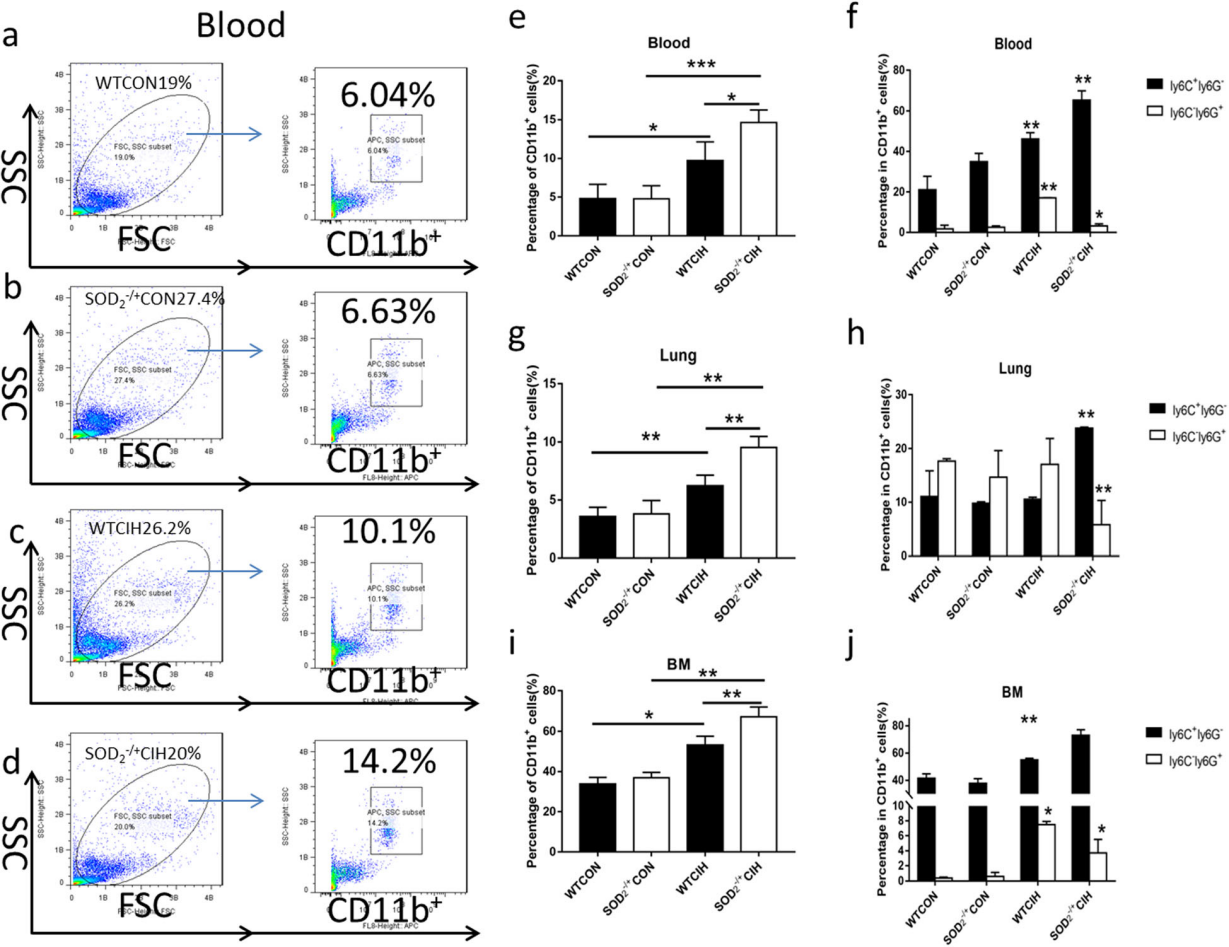
Flow cytometry analysis of percentage of CD11b^+^ cells in WT and SOD_2_^−/+^ mice. **a**-**d**. Representative view of flow cytometry graph of CD11b + cells in the blood of WT and SOD_2_^−/+^ mice. **e**. Percentage of CD11b^+^ cells in the peripheral blood mononuclear cells of the four groups analyzed by Flowjo software. **f**. Percentage of Ly6C^+^Ly6G^−^ cells and Ly6C^−^Ly6G^+^ cells in CD11b^+^ cells in the blood. **g**. Percentage of CD11b^+^ cells in the pulmonary mononuclear cells of the four groups. **h**. Percentage of Ly6C^+^Ly6G^−^ cells and Ly6C^−^Ly6G^+^ cells in CD11b^+^ cells in the lung. **i**. Percentage of CD11b^+^ cells in the bone marrow mononuclear cells of the four groups. **j**. Percentage of Ly6C^+^Ly6G^−^ cells and Ly6C^−^Ly6G^+^ cells in CD11b^+^ cells in the bone marrow. CON: Wild type mice in the normal air condition. CIH: Chronic intermittent hypoxia. WT: wild type mice. SOD_2_^−/+^: knock-down of SOD_2_ gene heterozygous mice. *N* = 5. Comparisons between multiple groups were performed using ANOVA with the Bonferroni test. *p < 0.05, **p < 0.01, ***p < 0.001

Given that SOD2 modulated CD11b+ cells proliferation or mobilization, we analyzed the percentage of CD11b+ cells in the lung, blood and bone marrow. Results showed that percentage of CD11b+ cells was elevated in the blood, the lung as well as the bone marrow by flow cytometry analysis (Fig. 4a-j, e, g, i), which demonstrated that CD11b+ cells were largely proliferated in bone marrow and mobilized to the lung and peripheral blood under the condition of SOD2 deficiency. Among CD11b+ cells, monocytic myeloid cell line-Ly6C+Ly6G-cells were increased, while granulocytic myeloid cell line-Ly6C-Ly6G+ cells were decreased (Fig. 4f, h, j).

**Fig. 5:**
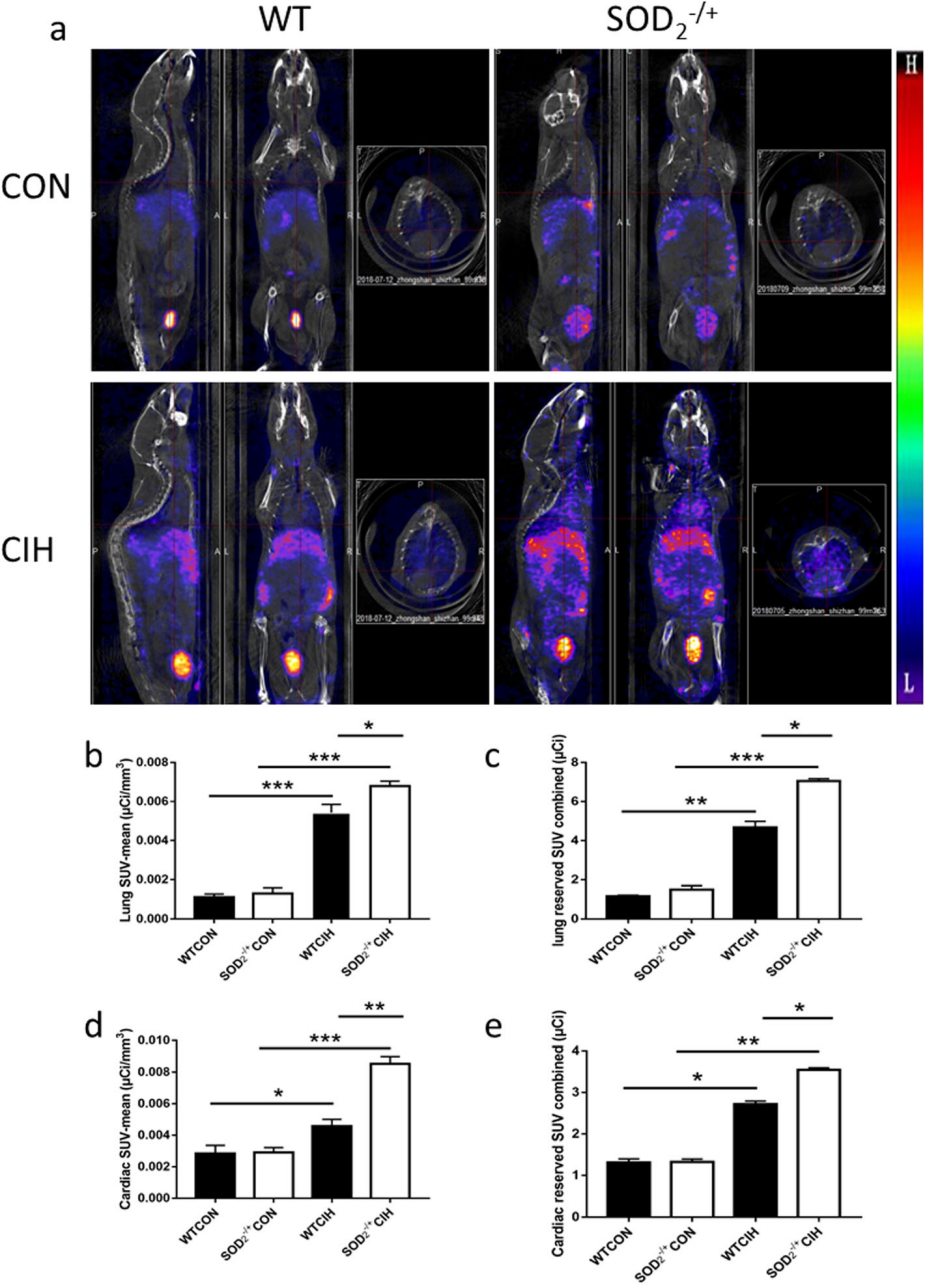
Micro-PET scanning of in vivo ^99m^Tc-labelled- MAG3-anti-CD11b in SOD2 deficiency mice. **a**. Representative radioactive heat map of WT and SOD_2_^−/+^ mice under condition of normal air and CIH. **b**. Lung mean-SUV value in the four groups. **c**. Total reserved SUV in the lung of WT and SOD_2_^−/+^ mice after CIH intervention. **d**. Cardiac mean-SUV value in the four groups. **e**. Total reserved SUV in the heart of WT and SOD_2_^−/+^ mice after CIH intervention. CON: Wild type mice in the normal air condition. CIH: Chronic intermittent hypoxia. WT: wild type mice. SOD_2_^−/+^: knock-down of SOD_2_ gene heterozygous mice. *N* = 5. Comparisons between multiple groups were performed using ANOVA with the Bonferroni test. *p < 0.05, **p < 0.01, ***p < 0.001

**Fig. 6:**
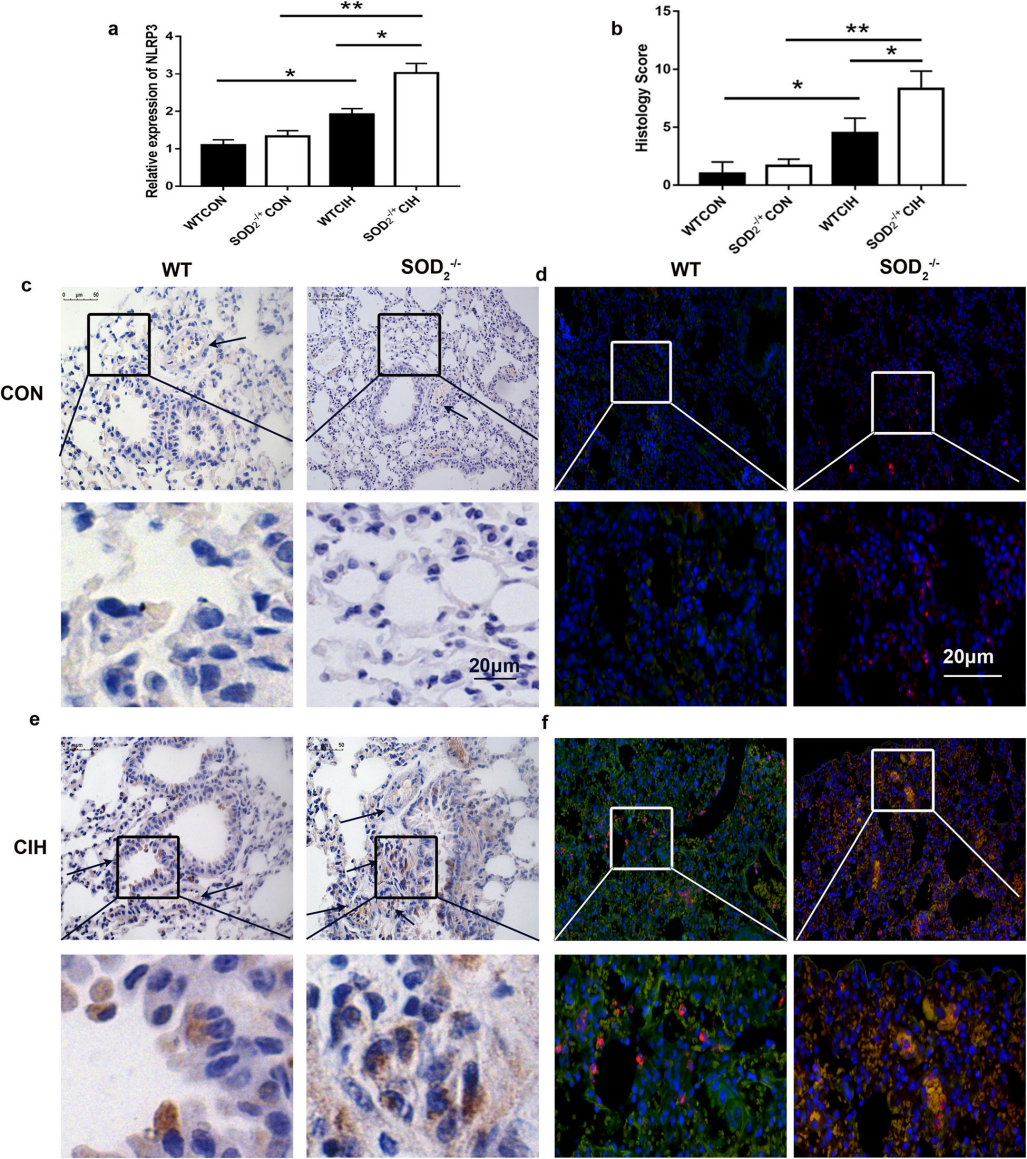
Expression of NLRP3 in PH and CIH model. **a**. mRNA expression of NLRP3 in the lung in WT and SOD_2_^−/+^ mice under normal air and CIH condition. **b**. Measurement of histology score of IHC staining of NLRP3 in the lung in the four groups. **c** and **e**. IHC staining of NLRP3 in the lung in the four groups. Arrows were pointed to the pulmonary arteriole. The rectangular frame was amplified in the same times in the picture of **c** and **e**; **d** and **f**. Co-staining of CD11b and NLRP3 by immunofluorescence in the four groups. Blue represented DAPI. Red represented NLRP3. Green represented CD11b. The rectangular frame was amplified in the same times in the picture of d and f. CON: Wild type mice in the normal air condition. CIH: Chronic intermittent hypoxia. WT: wild type mice. SOD_2_^−/+^: knock-down of SOD_2_ gene heterozygous mice. *N* = 5. Comparisons between multiple groups were performed using ANOVA with the Bonferroni test. *p < 0.05, **p < 0.01, ***p < 0.001

In vivo, MAG3-anti-CD11b was adopted to analyze the macroscopic distribution of CD11b+ cells in WT and SOD2 deficiency mice under CIH condition. Micro-PET scanning of in vivo 99mTc-labelled-MAG3-anti-CD11b showed that elevated statistically lung mean-SUV value and total reserved SUV in the lung of SOD2-/+ mice (Fig. 5a and Fig. 6c), which indicated more CD11b+ cells mobilized to the lung. Furthermore, more 99mTc accumulation in the heart of SOD2 deficiency mice compared to WT mice under CIH condition (Fig 5d-e). Taken together, CD11b, as an inflammatory marker, indicated deeper inflammatory injury under CIH in SOD2 deficiency mice. The visual change using radioactive marker is expected to be a biomarker in vivo to measure the degree of CIH related PH.

### NLRP3+CD11b+ cells involved in SOD2 associated CIH induced PH

Deficiency of SOD2 aggravated CIH induced PH. Previous study revealed elevated activation of NLRP3 in PH and CIH model. We next indicated that NLRP3 was increased in SOD2 deficient mice under CIH condition. Figure 6a showed increased mRNA expression of NLRP3 in the group of SOD2-/+ after CIH. Figure 6b showed the measurement of histology score of IHC staining of NLRP3 in the group of SOD2-/+ was higher than the group of WT mice. IHC staining of NLRP3 in the lung showed that NLRP3 was mainly expressed in interstitial cells of the lung (Fig. 6c, e). Co-staining of CD11b and NLRP3 indicated that NLPR3 was mainly expressed in CD11b+ cells. Comparing to the group of WT mice, NLPR3+CD11b+cells was increased in the group of SOD2 deficiency mice under CIH condition (Fig. 6d, f). Thus, we put forward that SOD2 in CD11b+ might regulate CIH induced PH through NLRP3 pathway.

### SOD2-deficient-CD11b+ myeloid cells promoted the apoptosis resistance and over-proliferation of human pulmonary artery smooth muscle cells (PASMCs) via up-regulating NLRP3

**Fig. 7:**
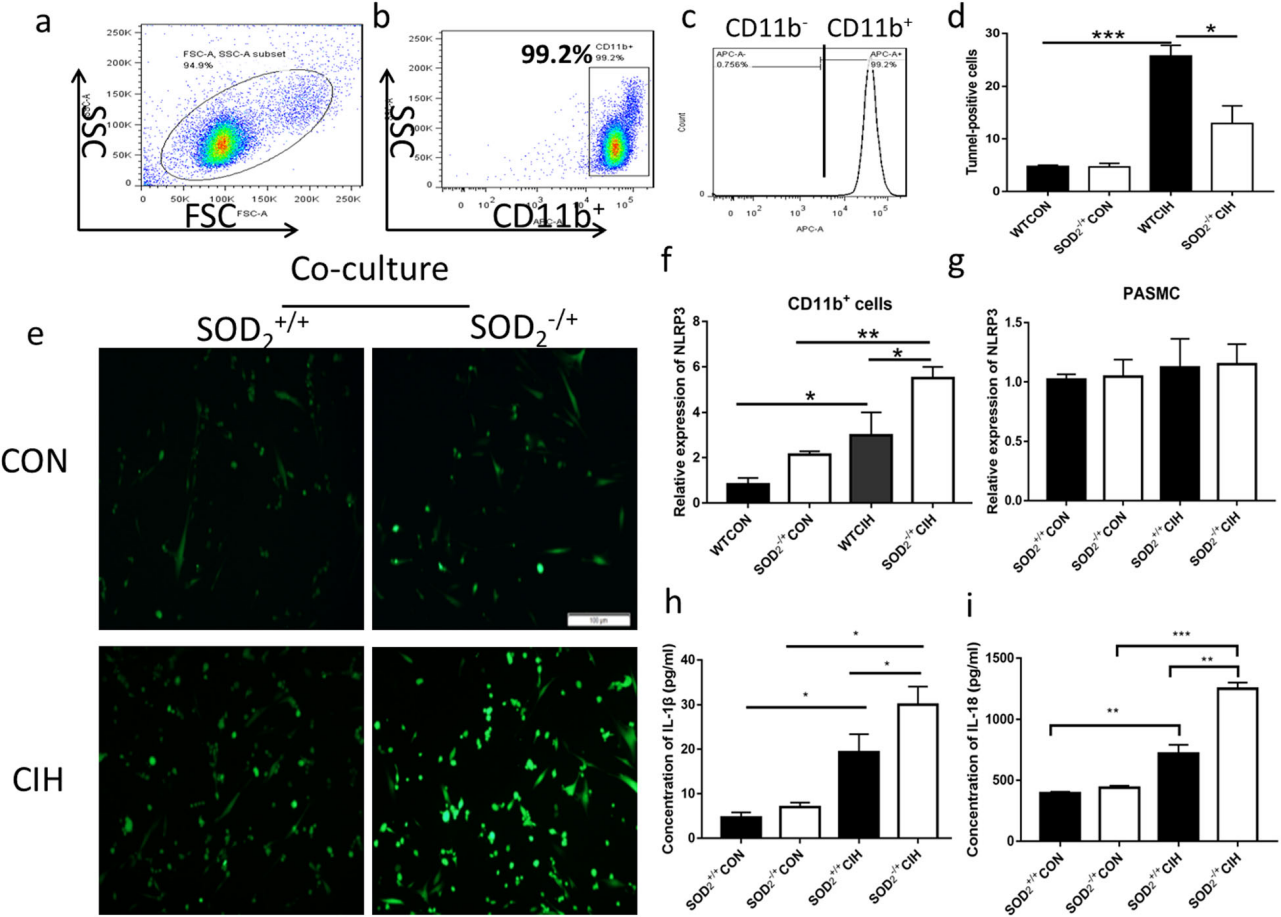
Co-culture CD11b^+^ myeloid cells and PASMCs. Co-culturing SOD_2_-deficient-CD11b^+^ myeloid cells and PASMC cells using transwell plates (CD11b^+^ cells in the upper side; PASMCs in the lower side). **a**-**c**. Separation gain rate of CD11b^+^ cells analyzed by FACS. CD11b^+^ cells were separated from C57BL/6 and SOD_2_^−/+^ mice using magnetic beads. **d**. PASMCs (the lower side plates) were stained to measure the percentage of tunnel positive cells. **e**. Brdu positive staining in PASMCs in the four groups. **f**. mRNA expression of NLRP3 in CD11b^+^ cells. **g**. mRNA expression of NLRP3 in PASMCs. **h**. Secretion of IL-1β in the supernatant of the co-culture system. **i**. Secretion of IL-18 in the supernatant of the co-culture system. CON: Wild type mice in the normal air condition. CIH: Chronic intermittent hypoxia. WT: wild type mice. SOD_2_^−/+^: knock-down of SOD_2_ gene heterozygous mice. *N* = 5. Comparisons between multiple groups were performed using ANOVA with the Bonferroni test. *p < 0.05, **p < 0.01, ***p < 0.001

How do these elevated CD11b+ cells act on PASMC cells? We separated CD11b+ cells from bone marrow of SOD2 deficiency mice and WT mice under normal air and CIH condition using anti-CD11b magnetic beads. The CD11b+ cells gain rate was beyond 99% (Fig. 7a-c). To further ascertain the role of SOD2-deficient-CD11b+ cells involved in CIH induced PH model, we co-cultured SOD2-deficient-CD11b+ myeloid cells and PASMC cells using transwell plates (CD11b+ cells in the upper side and PASMCs in the lower side). Marked reduction of tunnel positive PASMCs after co-culture with SOD2-deficient-CD11b+ myeloid cells compared to the group of SOD2+/+ -CD11b+ cells (Fig. 7). Under condition of co-culture with SOD2-deficient-CD11b+ cells, Brdu positive cells were increased in PASMC cells compared with control (Fig. 7e). Expression of mRNA of NLRP3 in these CD11b+ cells and PASMCs were analyzed. It was further verified that NLRP3 was elevated in SOD2-deficient-CD11b+ myeloid cells but not in PASMCs (Fig. 7f-g). Secretion of IL‐1β and IL-18 were increased in the supernatant following the endogenous NLRP3 protein activation (Fig. 7h, i). Activation of the NLRP3 inflammasome is well known to be associated with the oxidative stress. The results indicated that SOD2-deficient-CD11b+ myeloid cells could activate NLRP3 inflammasome, aggravate proliferation of PASMC, and promote release of IL‐1β and IL-18, which led to the apoptosis resistance and over-proliferation of PASMC.

## Discussion

**Fig. 8:**
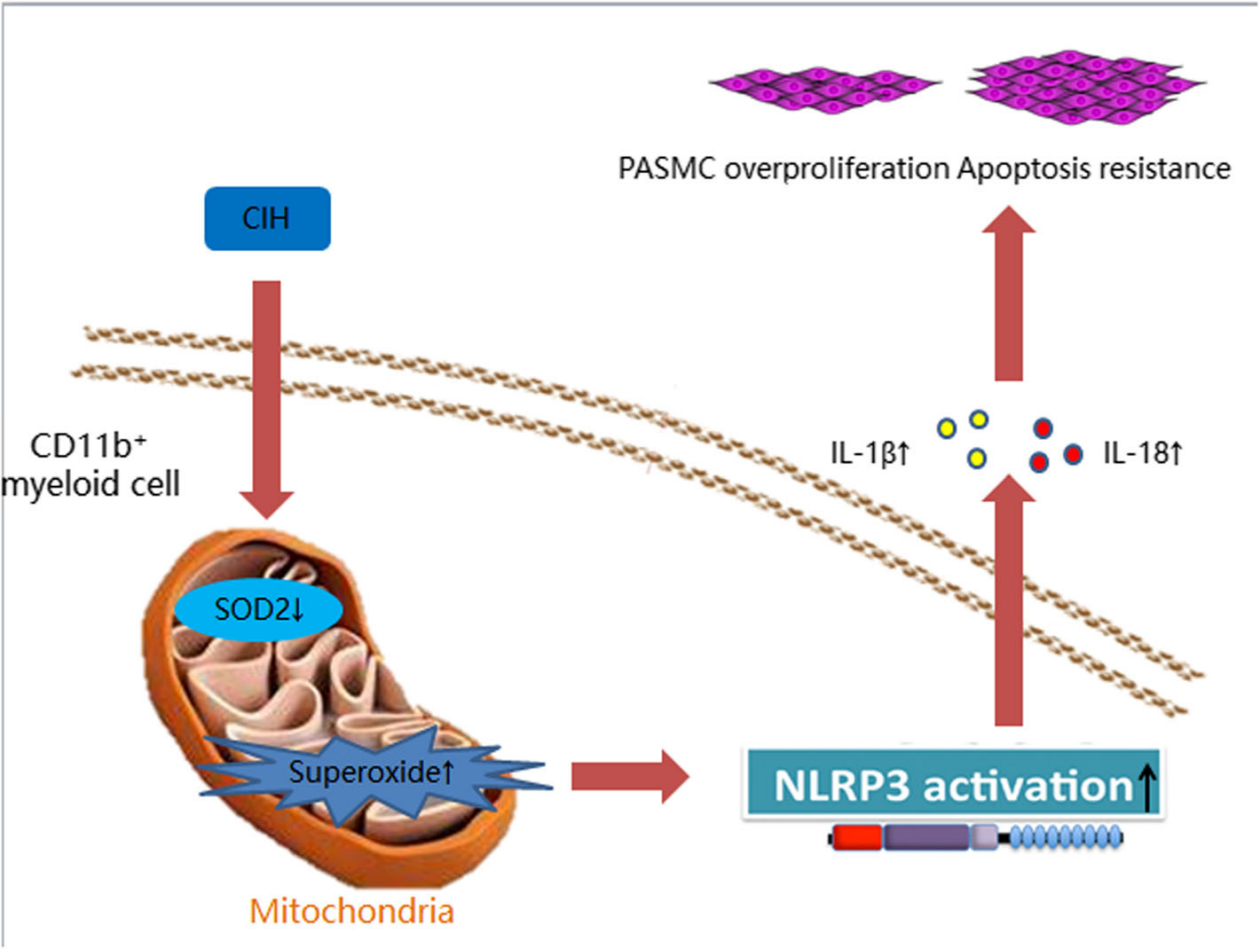
Flow chart of the role of SOD2 in chronic intermittent hypoxia induced pulmonary hypertension.

This study revealed that expression of SOD_2_ is a pivotal molecular in the pathophysiological process of CIH induced PH. Our study showed that knockdown of SOD_2_ increased pulmonary hypertension and vascular muscularization. CIH induced lowering expression of SOD_2_ increased CD11b^+^ cells gathering in the lung and heart, activated NLPR3 inflammasome in CD11b^+^ cells, promoted IL-1β and IL-18 secretion, and aggravated PASMCs proliferation and resisted apoptosis. The mechanism flow chat was showed in Fig. 8.

Studies showed that CIH induced HIF-1α potentiates inhibitory functions of myeloid cells and is involved in driving the polarization towards M2-like macrophages, which was crucial for enhancing immunosuppressive functions and led to chronic inflammatory environment [15, 26]. We observed a significant increase in CD11b^+^ cells in the bone marrow, lung and blood, which mainly resulted in a selective enhanced output of Ly6C^+^ monocytes. Deficiency of SOD2 amplified this CD11b^+^ cells induction. Our study revealed that CD11b^+^ myeloid cells were crucial in the inflammatory process of CIH induced pulmonary vascular remodeling, which indicated immune suppression response during the chronic phase of CIH related diseases. Given that SOD_2_ plays a central role in oxidative stress and CD11b + cells transformation and mobilization, this finding points to a potential underlying mechanism for CD11b^+^ cells dysfunction and the increased susceptibility to PH [27, 28]. CD11b^+^ cells could be neutrophils, macrophages, MDSCs or NK cells. Further investigations are definitely warranted to better understand myeloid immune suppression.

Intracellular SOD_2_ has a protective role by suppressing NLRP3 inflammasome-caspase-1-IL-1β axis under inflammatory conditions [27]. Recent evidence shows that NLRP3 is involved in pulmonary vasoconstriction and progressive pulmonary hypertension [18]. The NLRP3 inflammasome comprising the apoptosis speck-like protein plays a key role in innate immunity and lung injury [29]. In particular, the NLRP3 pathway leads to inflammation and vascular remodeling, thus contributing to the development of PH [30]. Interestingly, mice lacking NLRP3 display attenuated pulmonary hypertension and a blunted inflammasome activation with decreased caspase-1, IL-18, and IL-1β levels in response to hypoxia induced pulmonary vasoconstriction [31, 32]. The mature IL-1β is a prototypic multifunctional cytokine that is involved in pulmonary inflammation and could stimulate chemokines and adhesion molecules, such as MCP-1 and MIP-1α for macrophage recruitment [33, 34], which contributed to CD11b^+^ cells mobilization in feedback.

We provided evidence supporting that deficiency of SOD2 activated NLRP3 and increased IL-1β secretion. It has been reported that in OSA, the expression of IL-1β increased and the NF-kB pathway was activated, along with endothelial dysfunction induced by hypoxia/normoxia, all of which are synergistic with the formation and development of OSA [35]. Reduced IL-1β expression alleviated PH in OSA, the mechanism of which was involved with inhibited HIF1 transcriptional activity and the NF-κB signaling pathway [36]. Moreover, reduced mitochondria-derived reactive oxygen species (ROS) production and increased SOD-2 activity, resulting in the suppression of NLRP3 inflammasome-mediated IL-1β secretion. Induction of IL-1β induced ROS generation and MMP-2 activation and promoted PASMCs proliferation [37]. Reduced mitochondria-derived ROS production and increased SOD-2 activity, resulting in the suppression of NLRP3 inflammasome mediated IL-1β secretion. Data also showed that augmented expression of IL-18 may perpetuate an inflammatory milieu that eventually contributes to the vascular obstruction characteristic of PH [38]. Deficiency of SOD_2_ in CD11b^+^ cells co-cultured with PASMCs in this study resulted in increased IL-1β and IL-18, which could be the direct efficient cytokine acting on PASMCs proliferation.

## Conclusion

CIH activated oxidative stress and reduced expression of SOD2 in mice. Deficiency of SOD2 increased CD11b^+^ cells gathering in the lung and heart, which activated NLPR3 inflammasome, promoted IL-1β and IL-18 secretion, aggravated PASMCs proliferation and resisted apoptosis. CIH induced down-regulating of SOD2 increased pulmonary hypertension and vascular muscularization. It could be the mechanism of CIH leading to PH.

## Supplementary information

**Additional file 1: Figure S1.** Representative RVSP wave in SOD_2_^-/+^ and WT mice by exposure to CIH for 6 weeks. **Figure S2.** Medial vascular wall thickness showed by elastica van gieson staining in the group of WT mice and SOD_2_^-/+^ mice under CIH condition. a. Representative image of elastica van gieson staining of mice in the four groups. b. Statistical results of vascular thickness of the four groups. *N* = 5. *p < 0.05, **p < 0.01, ***p < 0.001.

## Data Availability

The datasets used and/or analysed during the current study are available from the corresponding author on reasonable request.

## Abbreviations

(CIH): chronic intermittent hypoxia
(FACS): Fluorescence activating cell sorter
(H&E): Hematoxylin–Eosin
(IHC): Immunohistochemical
(NLRP3): Nod-like receptor pyrin domain containing 3
(OSA): Obstructive sleep apnea
(PASMCs): Human pulmonary artery smooth muscle cells
(PET): Positron emission tomography
(PH): Pulmonary hypertension
(ROS): Reactive oxygen species
(RT-PCR): Real time-polymerase chain reaction
(RV): Right ventricle
(RVCSA): Right ventricle cross-sectional area
(SDB): SLEEP disordered breath
(SOD2): Superoxide dismutase 2
(TAPSE): Tricuspid annular plane systolic excursion
(WT): Wild type
