# Supplementary material for: SOD2 ameliorates pulmonary hypertension in a murine model of sleep apnea via suppressing expression of NLRP3 in CD11b+ cells
Source: Respir Res. 2020 Jan 8;21:9. doi: 10.1186/s12931-019-1270-0 (PMC6951024; doi:10.1186/s12931-019-1270-0)

Figure 1s Representative RVSP wave in SOD_2_^-/+^ and WT mice by exposure to CIH for 6 weeks.


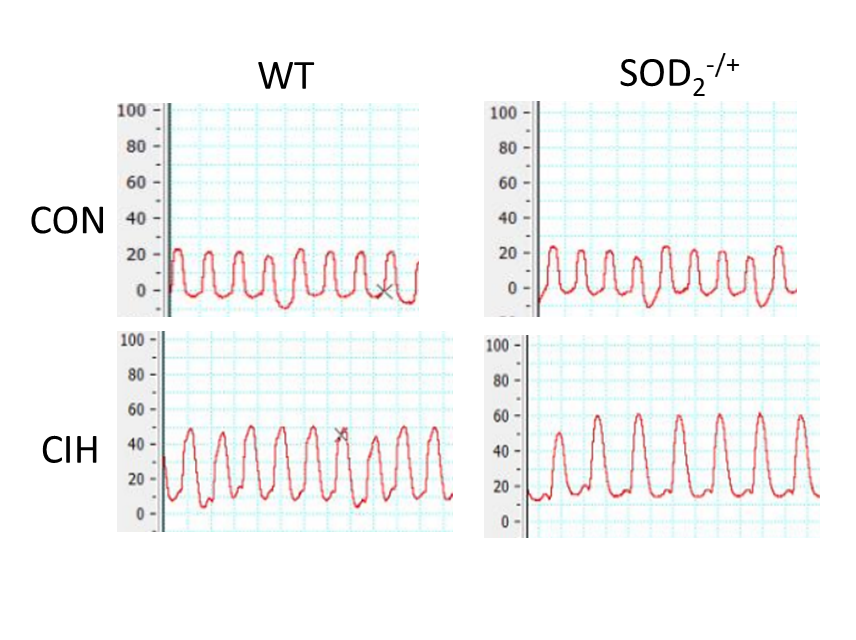


Figure 2s Medial vascular wall thickness showed by elastica van gieson staining in the group of WT mice and SOD_2_^-/+^ mice under CIH condition. a. Representative image of elastica van gieson staining of mice in the four groups. b. Statistical results of vascular thickness of the four groups. N=5. *p < 0.05, **p < 0.01, ***p < 0.001.


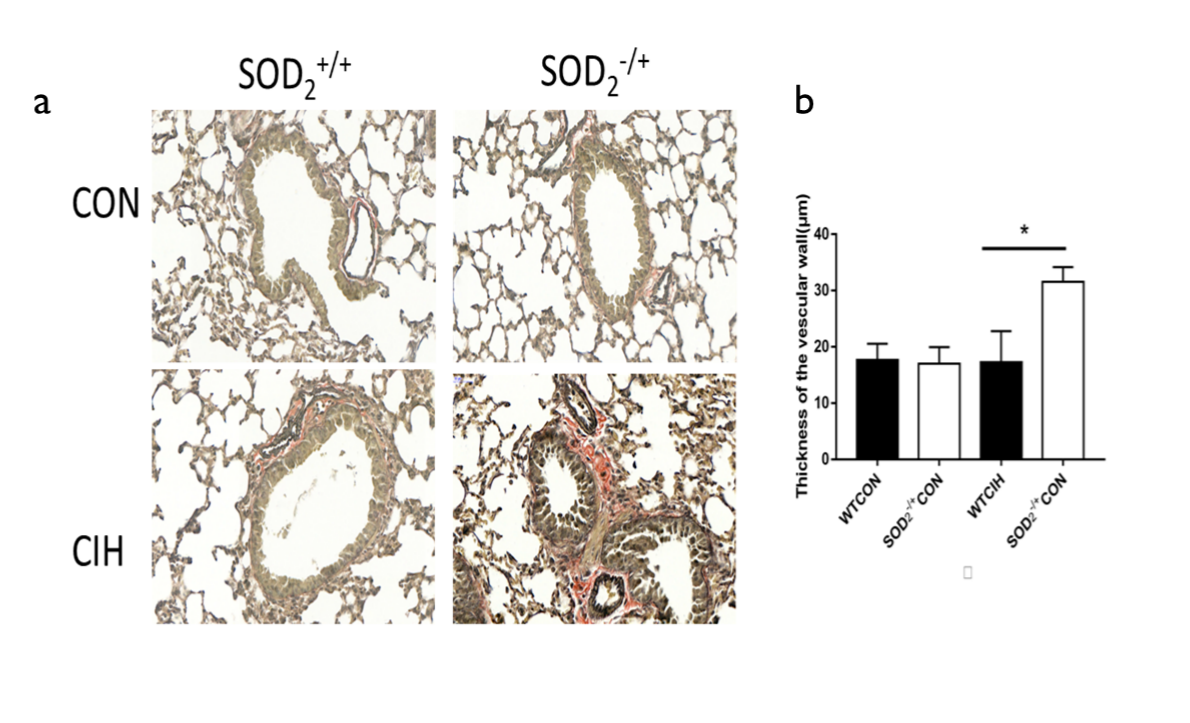

Supplement: Supplementary file 1 — Additional file 1: Figure S1. Representative RVSP wave in SOD2-/+ and WT mice by exposure to CIH for 6 weeks. Figure S2. Medial vascular wall thickness showed by elastica van gieson staining in the group of WT mice and SOD2-/+ mice under CIH condition. a. Representative image of elastica van gieson staining of mice in the four groups. b. Statistical results of vascular thickness of the four groups. N = 5. *p < 0.05, **p < 0.01, ***p < 0.001. [file 12931_2019_1270_MOESM1_ESM.docx]
